# An Instrument for the Characterization and Calibration of Optical Sensors

**DOI:** 10.3390/s21155141

**Published:** 2021-07-29

**Authors:** Enrico Gastasini, Niccolò Capecci, Francesco Lupi, Alessio Gagliardi, Sergio Saponara, Michele Lanzetta

**Affiliations:** 1 Alkeria Srl, Via Mario Giuntini 25, 56021 Cascina, Italy; e.gastasini@gmail.com (E.G.); n.capecci@gmail.com (N.C.); 2 Department of Information Engineering, University of Pisa, Via G. Caruso 16, 56127 Pisa, Italy; alessio.gagliardi@phd.unipi.it (A.G.); sergio.saponara@unipi.it (S.S.); 3 Department of Civil and Industrial Engineering, University of Pisa, 56126 Pisa, Italy; lanzetta@unipi.it

**Keywords:** optical apparatus, camera testing, light uniformity, CCD, CMOS, EMVA 1288 standard, visual inspection

## Abstract

This paper presents the development of a hardware/software system for the characterization of the electronic response of optical (camera) sensors such as matrix and linear color and monochrome Charge Coupled Device (CCD) or Complementary Metal Oxide Semiconductor (CMOS). The electronic response of a sensor is required for inspection purposes. It also allows the design and calibration of the integrating device to achieve the desired performance. The proposed instrument equipment fulfills the most recent European Machine Vision Association (EMVA) 1288 standard ver. 3.1: the spatial non uniformity of the illumination Δ*E* must be under 3%, and the sensor must achieve an f-number of 8.0 concerning the light source. The following main innovations have achieved this: an Ulbricht sphere providing a uniform light distribution (irradiation) of 99.54%; an innovative illuminator with proper positioning of color Light Emitting Diodes (LEDs) and control electronics; and a flexible C# program to analyze the sensor parameters, namely Quantum Efficiency, Overall System Gain, Temporal Dark Noise, Dark Signal Non Uniformity (DSNU1288), Photo Response Non-Uniformity (PRNU1288), Maximum achievable Signal to Noise Ratio (SNRmax), Absolute sensitivity threshold, Saturation Capacity, Dynamic Range, and Dark Current. This new instrument has allowed a camera manufacturer to design, integrate, and inspect numerous devices and camera models (Necta, Celera, and Aria).

## 1. Introduction

Cameras are essential systems in human-machine interactions because vision provides about 80% of the necessary information for life. This paper deals with evaluating the electronic response of images or, more generally, light or camera sensors. These optical sensors are compact devices capable of analysis using a receptor. An optical transducer converts light or photons into an electrical signal [1,2]. These include linear and area or matrix sensors, regardless of the working principle, e.g., Charge Coupled Device (CCD) and Complementary Metal Oxide Semiconductor (CMOS) [1,3]. Individual sensors and fully assembled products and devices integrating the mentioned image or light sensor are considered.

**Figure 1:**
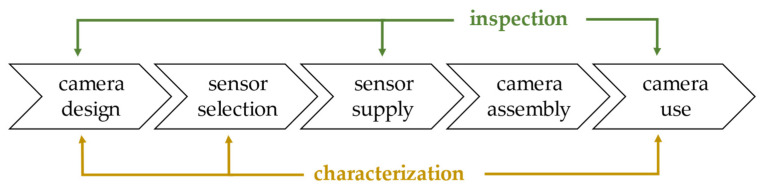
Main phases of the camera development process, showing the possible points of characterization and inspection (also for calibration purposes) by our instrument.

A stable, accurate, and consistent evaluation of the electronic response of a light sensor is required both for inspection (go/no-go testing) and for the characterization and correction of its response at different phases of the integrating device development process, as summarized in Figure 1. Optical sensors are the main component of a camera. For brevity, without loss of generality, we will refer to a camera as a typical product integrating an optical sensor in the remainder.

Such devices have a potential market among sensor manufacturers, sensor integrators, and final users of the integrated product. As shown in Figure 1, the characterization of the response of a sensor is, for example, required at the design stages, both of the individual sensor and of the integrating product. 

Inspection or quality assessment can be considered as a sub aspect of the sensor characterization. In this activity, the actual response is compared to the given specification set a priori. As shown in Figure 1, inspection is required in several stages of the sensor life:

- As an individual component during the sensor manufacturing for process control or before delivery.
- During the manufacturing of the integrated product (e.g., industrial, professional, mobile cameras), after supply by acceptance testing or sampling, at various assembly stages, and final testing.
- By the system integrator (e.g., a vision system or an optical instrument) for the firmware/software development and the final testing of the integrated system.
- By the final user (e.g., a factory or a laboratory) for setup, maintenance, and periodic calibration.

During the development of a product integrating a sensor, its electronic response is required to develop the interfaces and the rest of the electronics. The characterization of the electronic response in different conditions is also helpful to achieve the desired sensor behavior [4,5,6].

The electronic response of a sensor is included as technical datasheets accompanying the product. Sometimes, it requires validation by the customer, e.g., in the case of low cost products, different batches coming from external manufacturing plants, or inconsistent production. Cameras having the exact same specification datasheets may behave completely differently when viewing the same scene. Datasheets can also be biased, showing only the best qualities and characteristics of the product. For example, it is common to indicate the number of bits of the onboard Analog to Digital Converter (ADC), even though in reality, only a subset of the available bits will be used. 

The technical analysis of the camera can be done by the device manufacturer unbiasedly by standardizing the test process, allowing to compare the camera performance with respect to noise, dark current, sensitivity, gain, linearity, and other parameters.

The test principle applied in the proposed device is well established and is called Photon Transfer Curve (or Photon Transfer Method/Technique). It is used by the Goddard Space Flight Center (GSFC) and NASA’s Jet Propulsion Labs (JPL) as well as by camera manufacturers. It is based on the principles of black box (or rather grey box) system identification [7]: stimulating the system under examination with inputs (light) and recording its outputs (the digital data). The Photon Transfer Method is also the theoretical basis of the European Machine Vision Association (EMVA) 1288 *standard* [8], for the identification of the parameters of optical sensors.

This work has been carried out in collaboration with Alkeria, a manufacturer of digital cameras for industrial and biomedical devices [9], interested in implementing the *standard* for their characterization. 

### 1.1. Literature

Over the past several decades, many imaging sensors based primarily on CCD or CMOS technology were developed. Datasheets provided by developers are usually written on their standards, and no universal figure of merit can be drawn from them for comparison purposes [10]. Most manufacturers of sensors for industrial cameras do not fully specify their characteristics or apply sophisticated measurement methods, leaving to camera developers ambiguities that arise during camera design and require meticulous checks.

Camera calibration is a fundamentally necessary technology in computer vision. This process is mainly based on recovering the internal (i.e., principal point, focal length, and aspect ratio) and external (i.e., translation and rotation) parameters of a camera [11]. It usually involves (i) taking images of some specially designed patterns with known metric geometry or with some unique structures; (ii) extracting the pattern features from the images; and (iii) estimating the camera parameters using constraints derived from the extracted features [11].

According to [12], spheres have been widely used in camera calibration because of their symmetry and visibility in any orientation; a system that can calibrate both linear and distortion coefficients simultaneously for sphere images based on camera calibration was presented. 

The instrument proposed in this paper includes a Light Emitting Diode (LED) lighting system inside an Ulbricht sphere [13]. This sphere can integrate the radiant flow generated and projects a uniform light on the sensor surface to be tested [14,15]. Laser sources are not suitable being a coherent light source, which cannot be diffused by multiple reflections in the mentioned sphere [6]. Two illuminators that apply different feeding methods for LEDs to determine the most appropriate technique have been developed and are compared below. 

Various approaches for driving LEDs are available in the literature, such as in alternate [16] and direct current [17,18]. An experimental comparison is also presented here. 

Other characterization tools have been proposed in the literature, such as [19], which ensures up to 97% of homogeneous irradiation, and is overcome by our instrument, which exceeds 99%. 

In our instrument standalone software has been developed versus MATLAB application for the measurement and data storage proposed available in [19]. In [20], measurements to assess the scattering parameters of commercial off-the-shelf (COTS) camera lenses are presented. The EMVA 1288 *standard* was applied by insufficient information is available about the construction and performance of the experimental setup. Similarly, in [10], an experimental setup and software environment for radiometric characterization of imaging sensors following the *standard* guidelines is described. An interesting estimation of the influences and impact of several parameters on geometric measurements using simulation is also provided. Some of the feature of the proposed instrument (e.g., sphere sizing and LED wavelength) are inspired by the principles of the *standard*, but insufficient information is available about the hardware components and for the instrument realization.

Commercial solutions are also available on the market. For example, in [21] a service to characterize the camera according to the *standard* is offered but no specifications are reported about the equipment. 

Despite the existing *standard* requirements, a real complete hardware and software system implementation seems not yet available. The main contribution of this paper is a systematic analysis of possible approaches and a validated conceptual solution to fill this research gap.

### 1.2. EMVA 1288 Standard

The EMVA 1288 *standard*, more briefly referred to just as “The *standard*” in the remainder of this paper, provides a unified method to measure sensors and cameras for their characterization and comparison [8].

The *standard* was promoted by a consortium of camera manufacturers and stems from creating a protocol to uniquely determine the significant functional parameters and thus simplify consumer choice. It provides the *standard* for measurement and presentation of specifications for machine vision sensors and cameras. In particular, release 3.1 covers monochrome and color digital cameras with linear photo response characteristics [8]. The sensor analysis is resolution independent [13]. The *standard* text, split into four sections, describes the mathematical model and parameters that characterize cameras and sensors: linearity, sensitivity, and noise for monochrome and color cameras; dark current; sensor array non uniformities; and defective pixel characterization.

The *standard* presents an overview of the required measuring setup that is not regulated, not to hinder progress and the ingenuity of the implementers. Finally, the camera being tested can be described by the mathematical model on which the *standard* is based. The general assumptions include:

- The sensor is linear, i.e., the digital output signal increases linearly with the number of photons.
- The number of photons collected by a pixel depends on the product of irradiance *E* (units W/m^2^) and exposure time *t_exp_* (units s), i.e., the radiative energy density E*t_exp_ at the sensor plane.
- All noise sources are wide sense stationary and white concerning time and space. In other words, the parameters describing the noise are invariant with respect to time and space.
- Only the total quantum efficiency is wavelength dependent. Therefore, the effects caused by light of different wavelengths can be linearly superimposed.
- Only the dark current is temperature dependent.

These assumptions represent the properties of an ideal camera or sensor. If the deviation is slight, the description is still valid, and the degree of variation from an ideal behavior can be described. However, if the deviation is too large, the characterization is meaningless since camera parameters deviate from one of these assumptions.

The mathematical model proposed by the *standard* can be summarized as follows: the photons reaching the sensor are converted into electrons according to a percentage given by the quantum efficiency η. Therefore, a disturbance is added in the form of electrons, not photo generated, but caused by thermal agitation in the sensor. Then the signal is amplified and sent to an ADC that introduces its own quantization quantization noise. Finally, the signal coming out of the ADC represents the digital image on which the probabilistic calculations can be performed.

## 2. Development of the Proposed Instrument 

**Figure 2:**
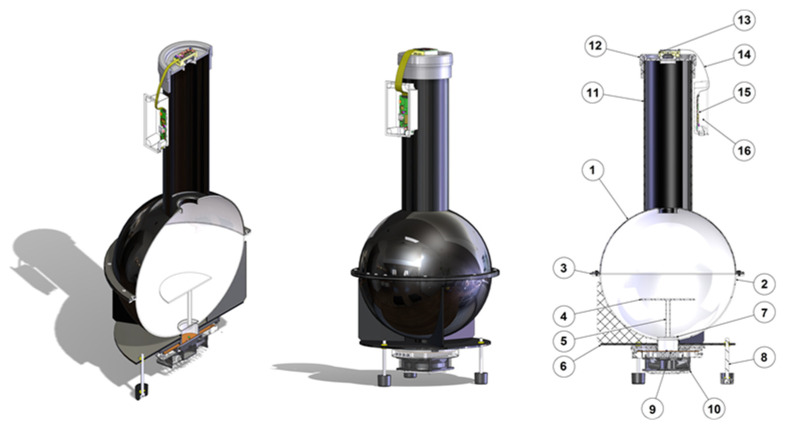
The rendered digital model of the proposed instrument in isometric view and main components: ①—Upper hemisphere. ②—Lower hemisphere. ③—Connection flange. ④—Deflector. ⑤—Support of the deflector. ⑥—Flange to support the sphere. ⑦—Separation glass. ⑧—Support feet. ⑨—LED illuminator. ⑩—Cooling system. ⑪—Dark tube. ⑫—Support for sensors/cameras. ⑬—Sensor/camera head. ⑭—Flat connection cable. ⑮—Sensor/camera Controlling Processing Unit (CPU). ⑯—CPU holder.

The proposed instrument was built around an integrating sphere, also known as an Ulbricht sphere [13]. The rendered Computer Aided Design (CAD) 3-dimensional (3D) model and constituent labelled elements is shown in Figure 2. This device has been designed to guarantee a light transmission with uniformity greater than 99%. For the verification the irradiance is measured at different points of the illuminated surface. 

The measurement system has the following main components and is described in more detail in the following sections.

- Sphere (Figure 2: ①–②): diffuses the light internally. Only the light rays perpendicular to the sensitive surface of the camera at the top of the tube (Figure 2: ⑪) can reach the sensor because the tube is coated internally with a non reflective material. More details are provided in Section 2.1. The experimental method to evaluate the light uniformity measurement is described in Section 4.
- Photodiode circuit (Figure 2: ⑬) to be inserted in the place of the camera to be tested to measure the number of photons reaching the sensor plane (detailed in Section 2.2).
- Illuminator with three Red, Green, and Blue (RGB) LEDs controlled via an FT232BL chip converting Universal Serial Bus (USB) to RS232 serial connection from the PC, detailed in (Section 2.3). The LEDs are placed in the lower part of the sphere (Figure 2: ⑨), opposite the sensor and thermally regulated by an air cooling system (Figure 2: ⑩).
- Control software on a Personal Computer (PC): controls the on/off switching of the individual LEDs of the illuminator, receives the grabbed images, processes images, and provides a report with numerical information, including the graphs required by the EMVA 1288 *standard* tests (Section 2.4).

### 2.1. Sphere

As for the construction of the hardware prototype, the sphere element (Figure 2: ①–②) has been one of the most challenging elements. Manufacturers’ datasheets and technical documents/procedures do not provide specific constructive indications, except a limit up to 5% for the openings vs total sphere surface ratio that guarantees light quality [15]; for the sphere dimensioning, a 1% ratio has been conservatively considered. Thus, the radius of the hemisphere has been determined accordingly. The two Polyvinyl Chloride (PVC) hemispheres have been internally and externally coated with standard white and black paint. This solution allows a uniform diffusion of the light generated by the illuminator without affecting the wavelength and prevents external disturbance. We discarded special barium sulphate paints for the internal coating due to the high cost of this solution. All the junction points have been carefully checked for perfect insulation from internal and external light interferences.

### 2.2. Photodiode

**Figure 3:**
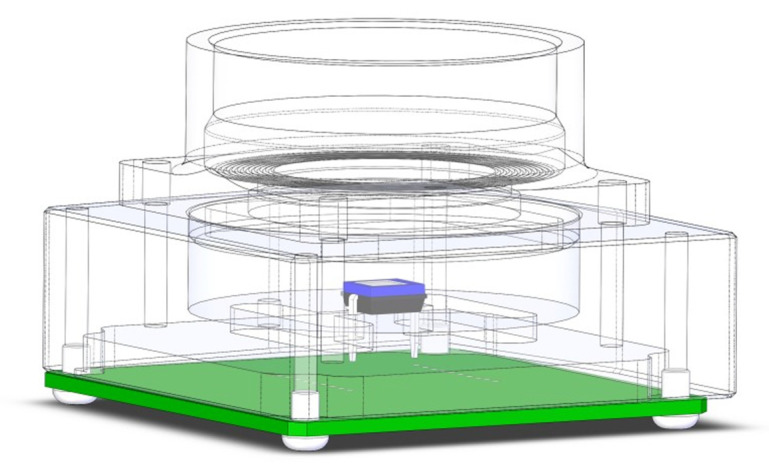
The photodiode in chassis.

**Figure 4:**
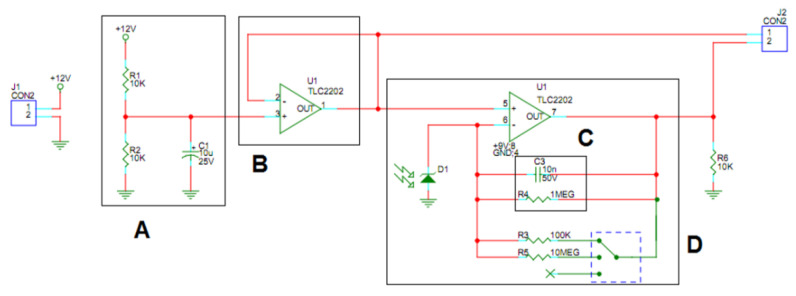
Photodiode conditioning circuit.

As for the electronic circuitry, the measurement system includes the photodiode drive circuit used for photon counting. The 3D CAD model of the chassis and conditioning circuit is shown in Figure 3, and its circuit is in Figure 4.

The Vishay BPW34 photodiode [22] is commonly used in commercial exposure meters because its response extends overall visible light up to the infrared. The photodiode has been installed on a current-voltage (I/V) conversion circuit made by an operational amplifier with a Field Effect Transistor (FET) as inputs. The Photonic Integrated Circuit (PIC) semiconductor integrated circuit acquires the voltage via a MAX11210 24-bit Maxim ADC.

The current generated by the photodiode (D1 in Figure 4) under illumination conditions is such that the dark current contribution, proper to the device, can be considered negligible. The circuit uses a Texas Instruments TLC2202 [23] dual low noise precision operational amplifier, ideal for low level signal conditioning applications. This amplifier is necessary to measure the output voltage proportional to the current of the photodiode that is detectable with an ordinary laboratory multimeter. The reverse current circulating in the photodiode can be derived by measuring this voltage. The irradiance (expressed in μW/cm^2^) can be thus calculated. Knowing the wavelength (and therefore the energy) of the individual photons emitted by the illuminator, it is possible to obtain the average value of the number of incident photons on each pixel of the sensor.

The resistors A in Figure 4 represent a voltage divider to halve the input voltage from 12 V to 6 V. A virtual ground and a buffer B in Figure 4 are used to lower its impedance. C in Figure 4 is a low pass RC filter with a time constant of about 0.01 s. The jumper D in Figure 4 is used to allow measurements in higher light conditions by reducing the sensitivity and resistance of the circuit. A third possibility is to leave the switch floating, so only the fixed 1 MΩ resistor remains in the circuit. 

### 2.3. Illuminator

**Figure 5:**
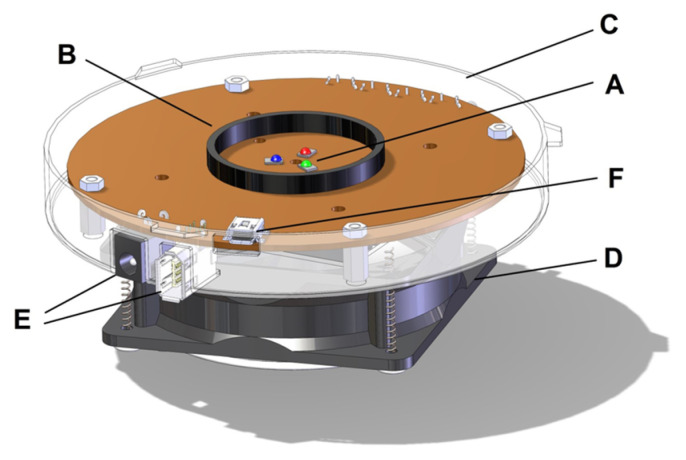
The main illuminator components: A—RGB LED. B—Tube to channel the light produced. C—Sphere attachment flange. D—Cooling system (fan). E—Power supply. F—USB socket for board control.

The other element of electronic circuitry is the illuminator. The one adopted presents a set of RGB LEDs. The rendered 3D CAD model image is shown in Figure 5.

The driving system of the illuminator consists of a PIC that receives commands from a PC via a USB-RS232 converter (F in Figure 5) and manages an AD7414 ADC. A variable current generator is controlled using a Metal Oxide Semiconductor Field Effect Transistor (MOSFET) and a feedback chain that keeps its output stable at a steady state through the ADC.

The software allows selectively turning on the three LEDs on the board and adjust the light intensity. The illuminator includes bracket C in Figure 5 for connection to the sphere. Tube B conveys the light produced (Figure 5: label B) and the fan D for thermal stability. 

### 2.4. Control Software

**Figure 6:**
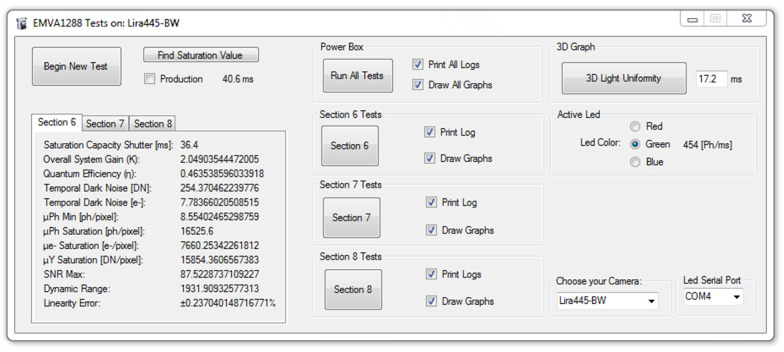
Example output of the main control software interface obtained during the tests of the Alkeria Lira445-BW camera.

The main interface of the program that supervises the instrument tests can be seen in Figure 6. The program can be used in two modes: characterization and inspection.

1. The camera characterization requires many samples, allowing accurate evaluation of the camera properties.
2. The inspection mode is used in production with lower testing time.

**Figure 7:**
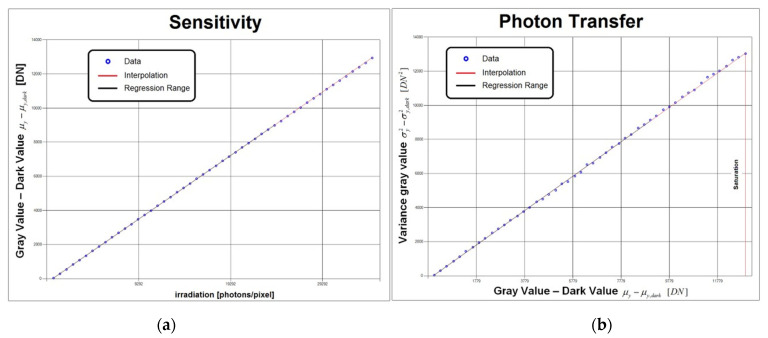
Example “Sensitivity” (**a**) and “Photon Transfer” (**b**) graphs were obtained for the Alkeria Lira 424 BW camera.

The software controls the illuminator and the camera under test as well as statistically processing the acquired images according to the EMVA 1288 *standard* (Section 1.2). The user can store the log and output the graphs of the tests (e.g., those in Figure 7). The *standard* recommends using a green light only. However, the other two LEDs can also be activated to operate a spectral analysis of the quantum efficiency defined by the formula η = R/K (where R and K are the angular coefficients of the Sensitivity and Photon Transfer graphs of Figure 7).

According to the developed Graphical User Interface (GUI), all the tests can be performed at once or, according to the *standard*, sequentially following the buttons labelled with the section numbers in Figure 6. In particular, the *Section 6* button (in Figure 6) represents the calculation of quantum efficiency, system gain, sensitivity testing, linearity, and temporal noise evaluation (example results are in Section 3). The *Section 7* button (in Figure 6) calculates the dark current (example results in Section 3). The *Section 8* button (in Figure 6) can be used for the calculation of spatial non uniformities of the sensor of the two indices proposed by the *standard*, Dark Signal Non-Uniformity (DSNU1288) and Photo Response Non Uniformity (PRNU1288) [8], discussed in Section 3 and Section 4.

The testing method follows Chapter 6 of the EMVA 1288 *standard* [8], which defines how the main parameters should be calculated.

## 3. Experimental

Fifty camera models have been developed using this instrument and its evolution over ten years. Sensors not compliant with the manufacturer’s blemish specifications have been rejected. Cold/hot pixels have been fixed, and unknown issues have been solved using a wide range of tests and analyses derived from the EMVA 1288 *standard*. According to the *standard*, the tests are carried out with fixed illumination and using the analysis mode, taking 50 sample images. 

**Figure 8:**
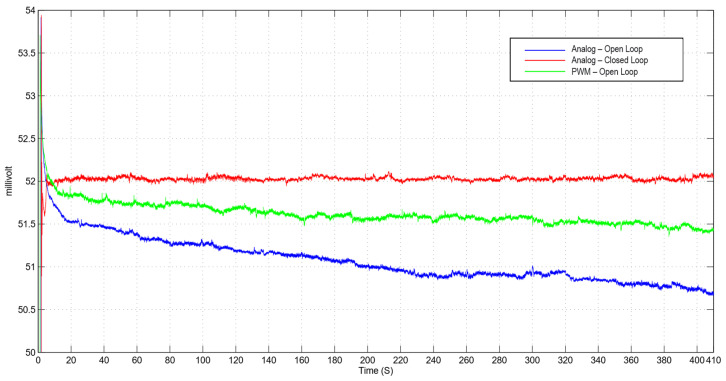
Comparison of the fan less thermal drift at steady state of three different driving modes of LEDs.

The camera operates at a constant temperature of 30 °C (measured on the chassis, Figure 3). A thermal chamber is unnecessary because the tests are completed in one hour, thus keeping the ambient temperature constant. The *standard* does not mandate the use of a climate chamber; the control software is designed to keep the LED on for about 150 s before running the tests to reach the thermal equilibrium. The light intensity dependence from the temperature has been experimentally investigated, as shown in Figure 8. The photodiode detector input from the analog illuminator in closed loop is stable as opposed to the decrease due to heating clearly shown in open loop. Exposure time vs lighting intensity has shown no linear deviations either for the Pulse Width Modulation (PWM) or the analog control.

The LED in PWM in open loop shows a slightly worse performance; however PWM is probably preferable for various reasons:

- it is more efficient from a thermal point of view thanks to the digital components that require less power;
- the system has simpler components than the analog board;
- it can be easily controlled remotely.

The open loop analog illuminator has a simple command interface and a limited noisy output. The power supply circuit requires current feedback to keep the current stable at a steady state. The increase in temperature leads to a decline in the conversion efficiency, which produces a decrease in the light output of the activated LED. 

The closed loop analogue illuminator solves the problem of brightness decay, compensating for the phenomenon through the feedback of the irradiance exiting the sphere. However, this method does not reduce power dissipation, which persists, and requires a fan to improve heat dissipation.

The complete tests on two camera models to describe the operation of the instrument in detail are presented: Alkeria Lira models 424 BW and 445 BW having a 1/3-inch CCD black and white sensor. Table 1 shows the results of an example of a test.

**Figure 9:**
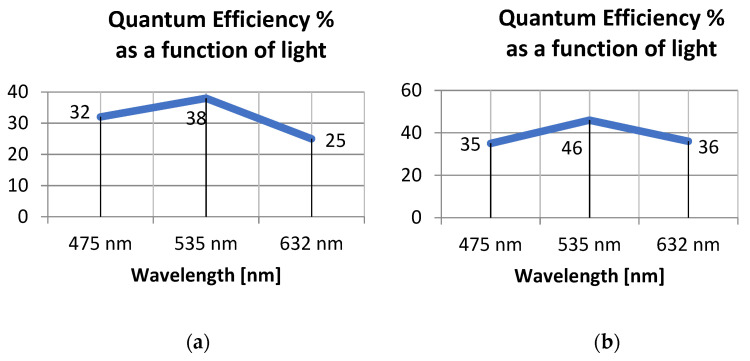
Example Quantum efficiency graphs of Alkeria Lira camera models 424 BW (**a**) and 445 BW (**b**).

Figure 9 presents the results of the spectral analysis applied to the quantum efficiency calculation. As expected, the peak efficiency occurs using a green light (at 535 nm wavelength).

## 4. Instrument Validation Method

**Figure 10:**
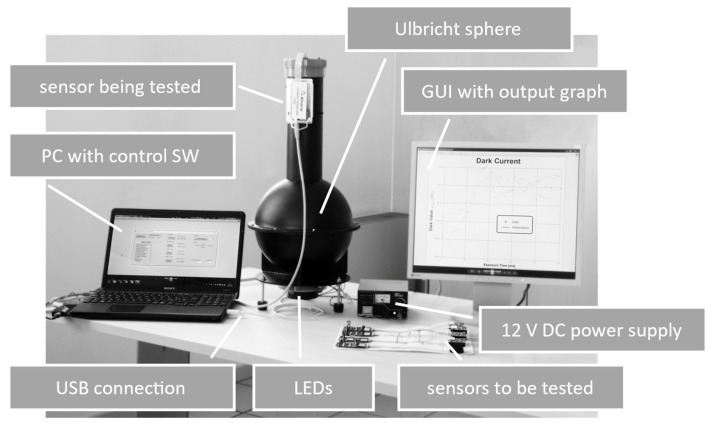
Experimental setup of the first instrument prototype with the main modules described in Section 2.

Figure 10 shows the configuration of the first instrument prototype developed.

**Figure 11:**
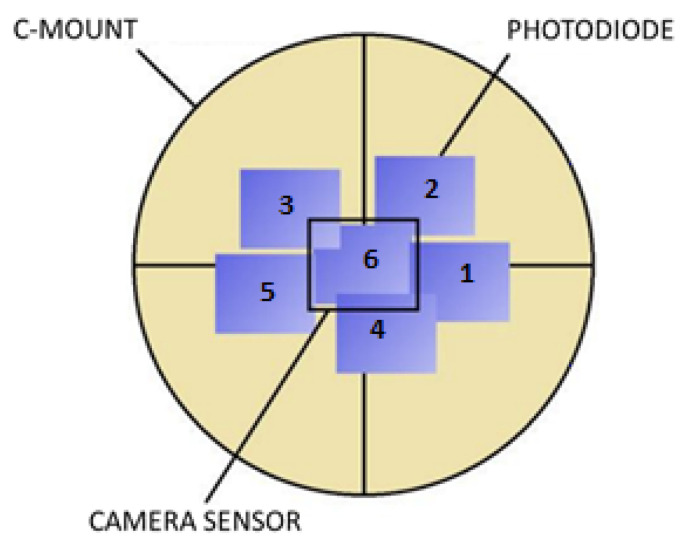
Photodiode positions for the irradiance uniformity measurement tests.

To measure the uniformity of the light irradiated on the sensor, we used the photodiode circuit described in Figure 4. The photodiode has been moved at six different positions. The six positions have been chosen around (1 to 5) and exactly coinciding (6) to the default camera/sensor position, as shown in Figure 11. The photodiode is placed at the same distance as the camera/sensor with respect to the output port of the sphere. 

**Figure 12:**
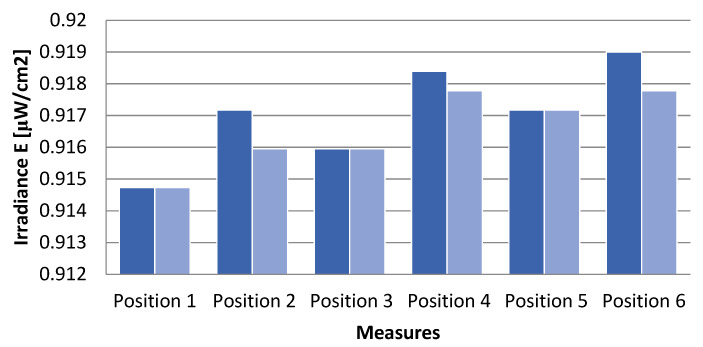
Light uniformity on the instrument measured with the photodiode described in Section 2.2. The results of two tests are shown in blue and light blue.

The measurements in Figure 12 have been carried out using a green LED with a current of 0.5 A. The current is set under the maximum to avoid overheating the illuminator and maintain a source of illumination with a more stable intensity. 

The sensor output has been sampled at 1 Hz and averaged for about 1 min at each position. Figure 12 shows the temporal stability during two tests.

The *standard* recommends not using a light source with a spatial disuniformity Δ*E* greater than 3%. The formula by which this value can be calculated is as follows:

(1) $$\Delta E\left[\%\right]=\frac{E_{\max}-E_{\min}}{\mu_{E}}\cdot 100$$

From the measurements in Figure 12, the instrument non uniformity is:

(2) $$\Delta E[\%]=\frac{0.919-0.9147}{0.9168}\cdot 100=0.469$$

Thereby, the uniform light can be calculated as 1 − Δ*E* = 99.53%.

**Figure 13:**
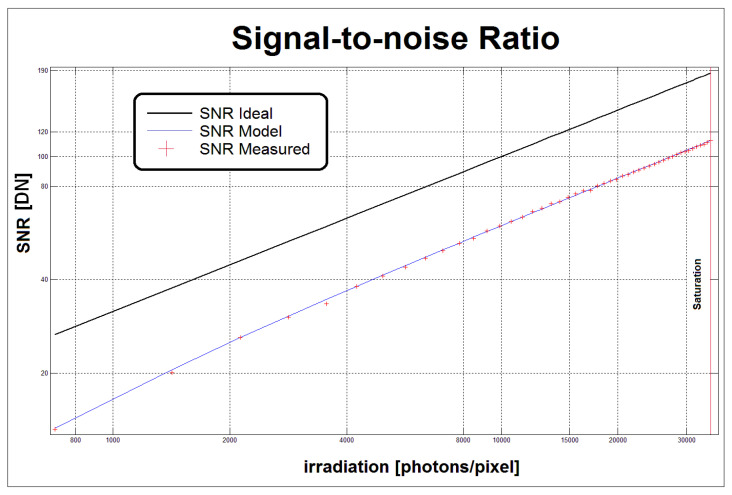
Signal to noise ratio of the Alkeria Lira camera model 445 BW.

Figure 13 shows the reliability of the proposed model: the values of the signal to noise ratio, represented by red crosses, are calculated from the grabbed images; the blue line shows the least squares approximation of the values calculated using some of the model parameters presented in Table 1. A Parameters Error (PE) index was created to check the variation of the SNR calculated from the model parameters vs the actual SNR. PE is calculated as a vector of percentage differences between the samples of the two SNRs, compared to full scale. The infinite norm is calculated from this vector. The PE represents the absolute value of the maximum percentage deviation between the actual SNR and that evaluated by the model parameters measured by the instrument. The PE index for the Lira 424 BW was on average 1.58%, while for the Lira 445 BW, it was 1.49%. Since the SNR is calculated from model parameters such as quantum efficiency, gain, dark current variance, and quantization noise, the non Linearity Error (LE) can be considered a good indicator of the correctness of the parameters measured by the instrument.

The estimated parameters of the Lira 424 BW are compared to the Basler scA640-70 gm camera, equipped with the same sensor: Sony ICX424-AL, to assess the results consistency. Unfortunately, the manufacturer tested the Basler camera using version 2.01 of the *standard*. No updated characterization is available vs the most recent *standard* considered in this work, so the values compared in Table 2 are indicative.

It can be noticed that the parameters in Table 2 are close, and the Alkeria model shows a better dynamic range and spatial non uniformity. This is because the linkage between the greater temporal stability and the dark current is instead caused by the measurement conditions. The metal casing of the Alkeria model is designed to dissipate the heat generated by the sensor control electronics on the metal bracket that connects it to the host machine. On the other hand, the instrument is made of plastic and does not dissipate heat, which is also not necessary for the measurement.

**Figure 14:**
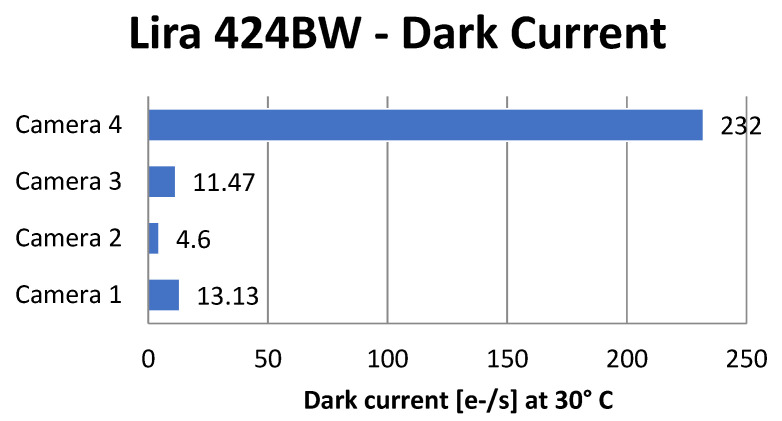
Dark current on four different Alkeria cameras model Lira 424 BW.

Figure 14 shows the dark current measurement for four different Alkeria cameras (1 to 4) of the same model Lira 424 BW. Camera 4 was produced 13 months earlier than the other three cameras, and, presumably, the Sony sensor used belongs to a different production batch. It can be noticed from the graph that the instrument was able to detect this variation in the Sony production process, which could not be appreciated otherwise.

**Figure 15:**
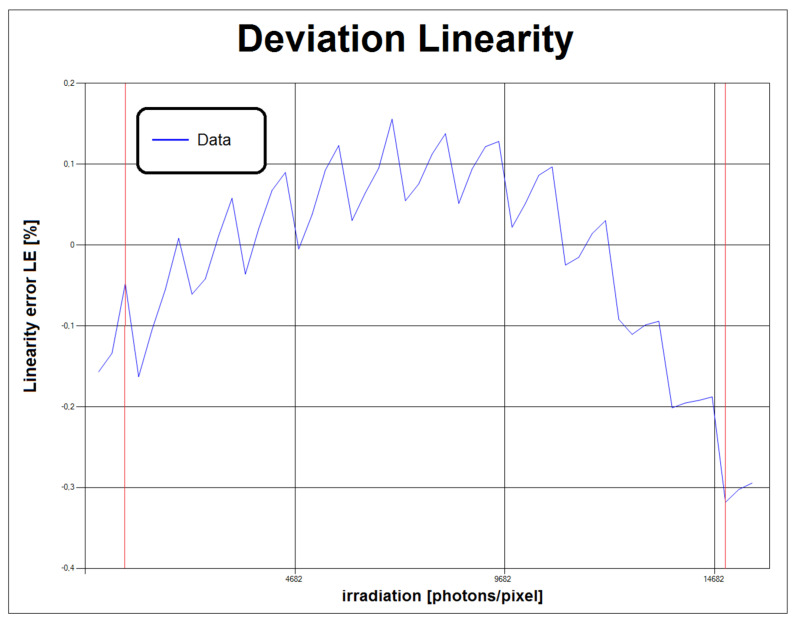
Graph generated by the control software of the instrument showing the effect of the exposure time implementation on the linearity error for the Alkeria Lira 445 BW camera. The vertical red lines highlight the minimum and maximum limits used by the regression (5–95% of the saturation value).

Figure 15 shows the graph of the percentage linearity error as a function of the quantity of photons reaching the sensor as a function of the effective exposure time (*t_esp_*) set on the camera. Supposing that the granularity of the exposure time setting is not a submultiple of the unit of measurement, a “sawtooth” trend results for *t_esp_*. This peculiar trend requires a firmware upgrade.

As for the inspection mode, deviations between cameras of the same model can be statistically analyzed; it has been established to reject devices with non linearity of more than 5% with respect to the average value for the same model. A similar criterion is extended to all the critical parameters in Table 1 for production quality improvement purposes. Similarly, the inspection of cameras returned for repair provides feedback for the quality improvement of the design in the interest of long term reliability. The analysis of this trend (Figure 15) reveals the unwanted behavior due to the implementation of the *t_esp_* control. The identified pattern (Figure 15) represents another remarkable result of the proposed system as it brings out a camera FW limit not easily verifiable with conventional techniques.

## 5. Conclusions

This paper has presented the design and the experimental evaluation of an innovative hardware/software system to characterize the electronic response of optical (camera CCD or CMOS) sensors.

Among the main innovations vs the state of the art are the design of an Ulbricht sphere providing a uniform light distribution fulfilling the EMVA 1288 *standard* ver 3.1; an innovative illuminator with proper positioning of RGB LEDs and control electronics as described; and a flexible C# program to analyze the sensor parameters also presented.

The paper has shown that using the proposed instrument. It is possible to carry out a complete and reliable characterization of the specific parameters of the sensor used. This allows a more informed choice of sensors based on their actual performance and calibration. The considered parameters indicated by the current *standard* ver 3.1 are the Quantum Efficiency, Overall System Gain, Temporal Dark Noise, DSNU1288, PRNU1288, SNRmax, Absolute sensitivity threshold, Saturation Capacity, Dynamic Range, and Dark Current.

The proposed instrument has been applied for ten years to characterize the integration and to inspect over fifty camera models by Alkeria. Validations have been carried out by direct measurement and indirectly by comparing data sheets of different sensors and similar camera models on the market.

Among the potential instrument improvements are:

- A calibration procedure for verifying the effects of hardware and software changes of the camera in real time. This can be achieved using an iterative testing method in the design phase that allows tweaking the hardware by quickly converging towards an optimal solution.
- Using a real radiometer instead of the Vishay photodiode would allow the certification of the results produced and greater accuracy in detecting the number of photons incident on the sensor.
- Extending the spectral analysis using a series of LEDs at different wavelengths or a broad spectrum illuminator with a bank of filters at set wavelengths, particularly for color sensors.
- Addition of a temperature sensor and control to increase the instrument productivity, speed up the LED warming, and prevent the risk of overheating. This would also allow characterizing the dependence of the dark current on the temperature.
- Scaling the instrument in size by a modular design accommodates the downsizing of sensors, e.g., by additive manufacturing for design and part replacement flexibility and checking the potential effect of geometric accuracy [24].

## Figures and Tables

**Table 1:** Test results for the Alkeria Lira 424 BW camera. Data are obtained, averaging four repetitions. Dimensionless units are explicitly declared according to the EMVA *standard* by the [DN] notation.

Data	Symbol	Typical	DevStd	Quantity
Quantum Efficiency ^1,2^	η	37.55	0.52	%
System Gain Inverse	1/K	1.0034	0.0149	e−/DN
Inverse of Photon Transfer	1/ηK	2.67	0.02	p^~^/DN
Temporal Dark Noise	$\sigma_{y,dark}^{2}$	157	24	DN^2^
	$\sigma_{d}$	12.57	1.12	e−
Dark Signal Non Uniformity	DSNU_1288_	0.8337	0.59	DN
Maximum achievable SNR ^3^	SNR_max_	121.2	0.88	DN
		41.67	0.06	dB
		6.92	0.01	bit
Inverse of Max Achievable SNR	SNR_max_^−1^	0.825	0.006	%
Photo Response Non Uniformity	PRNU_1288_	0.1966	0.04	%
Non Linearity Error	LE	±0.253	0.036	%
Absolute Sensitivity Limit ^1^	µ_e,min_	6.3	0.2	e−
	µ_p,min_	16.75	0.379	p^~^
Saturation Capability ^1^	µ_e,sat_	14688	212	e−
Saturation Irradiance ^1^	µ_p,sat_	39117	354	p^~^
Dynamic Range		2336	65.14	DN
		67.37	0.24	dB
	DR_bit_	11.19	0.04	bit
Dark Current		63	108	DN/s
		65	111	e−/s

^1^ λ = 535 nm; ^2^ FWHM = 35 nm, ^3^ Signal to Noise ratio (SNR).

**Table 2:** Comparing two different camera models equipped with the same sensor (Sony ICX424-AL) using our instrument based on the EMVA 1288 ver. 3.1 for the Alkeria model and those declared by the manufacturer for the Basler model based on ver. 2.01.

Parameters	Alkeria Lira 424 BW	Basler scA640-70 gm
Temporal Dark Noise σ_d_	12.57 [e−]	11 [e−]
Saturation Capacity	14688 [e−]	14000 [e−]
Absolute Sensitivity Limit	16.75 p^~^	25 p^~^
Dynamic Range	11.19 bit	10.3 bit
SNR_max_	41.67 dB	41.6 dB
DSNU_1288_	0.8365 [DN]	2.9 [DN]
PRNU_1288_	0.1966%	0.5%

## Data Availability

Not applicable.
